# Editorial: The Endocannabinoid System: Filling the Translational Gap Between Neuroscience and Psychiatry

**DOI:** 10.3389/fpsyt.2021.771442

**Published:** 2021-10-28

**Authors:** Danilo De Gregorio, Mirella Ruggeri, Sagnik Bhattacharyya, Marco Colizzi

**Affiliations:** ^1^Neurobiological Psychiatry Unit, Department of Psychiatry, McGill University, Montreal, QC, Canada; ^2^Division of Neuroscience, Vita-Salute San Raffaele University, Milan, Italy; ^3^Section of Psychiatry, Department of Neurosciences, Biomedicine and Movement Sciences, University of Verona, Verona, Italy; ^4^Department of Psychosis Studies, Institute of Psychiatry, Psychology and Neuroscience, King's College London, London, United Kingdom

**Keywords:** cannabinoid (CB) receptors, perinatal and neurodevelopmental abnormalities, psychosis and depressive disorder, addiction and substance use disorder, laboratory model, magnetic resonance imaging (MRI), cannabidiol (CBD), genetics

Translational research has become a priority in every branch of medicine. In psychiatry, it has taken on the goal of advancing our understanding of the neurobiological underpinnings of cognition, behavior, and emotion, and the pathophysiological mechanisms affecting such processes that lead to the development of mental disorders. If successful in identifying objective measures of psychopathology and biomarkers of disease states, translational research will have a tremendous impact on psychiatry, allowing the achievement of a biologically relevant nosology and the development of treatments based on individual characteristics, thus giving rise to the era of personalized treatments (1).

Widely used worldwide, cannabinoids have attracted much attention for their potential role in health and disease, especially in psychiatry (2). Translational research has significantly advanced our understanding of the neuropsychiatric effects of cannabinoids (3–6). Adopting a translational perspective, the Research Topic presented here brings together up-to-date knowledge of these fascinating and complex chemical substances and how they may modulate mental health.

A recent report from the National Institute on Drug Abuse (NIDA) drew attention to the risks that using tobacco, alcohol, or illicit drugs, and misusing prescribed medication during pregnancy can carry for the offspring (https://www.drugabuse.gov/download/18910/substance-use-in-women-research-report.pdf?v=b802679e27577e5e5365092466ac42e8). Such warning stems from the evidence that substances can pass easily through the placenta to reach the fetus. In their review, Navarrete et al. summarize the evidence regarding the specific effects of cannabis use during the prenatal and postnatal periods, indicating behavioral and neurobiological aberrancies that can potentially persist throughout childhood and adolescence, and increased risk for the development of psychiatric disorders later in life, especially affective and substance use disorders (SUD). In their review, Nashed et al. come to similar conclusions, highlighting the risks specifically associated with consumption of cannabis varieties with high content of delta-9-tetrahydrocannabinol (Δ-9-THC), the main psychoactive exogenous cannabinoid, that is believed to cross the placenta and impact development. High use of cannabis during pregnancy, underestimation of its risk, progressive increase in cannabis potency in terms of Δ-9-THC concentration over the last two decades, and long-lasting neurodevelopmental consequences have been reported. Such evidence made authors emphasize the need to accelerate our knowledge of the effects of cannabis exposure during pregnancy and lactation across the life span.

One potential explanation for the higher risk of SUD during adolescence and adulthood, when prenatally exposed to cannabis, is that exogenous cannabinoids may affect the neurodevelopment of the endocannabinoid system (ECS), disrupting neurotransmission in brain areas regulating reward and motivation, thus increasing vulnerability to subsequent substance use and addiction. In their gene-by-gene interaction study, Elkrief et al. find genetically determined susceptibility to problem drinking related to specific polymorphisms in the *CNR1* gene, the gene coding for the cannabinoid receptor type 1 (CB1) protein, and other genes involved in endocannabinoid metabolism. Altogether, evidence points in the direction of alterations of the ECS, both genetically determined and environmentally induced, in the development of addictive behaviors. Of further interest, Pallanti et al. discuss the hypothesis that the ECS is crucial not only to *substance* addiction but also to *behavioral* addiction such as gambling disorder, now counted in the DSM-5 “substance-related and addictive disorders” section to highlight common biobehavioral underpinnings with SUD. To date, there is interest in the possibility that cannabidiol (CBD), the most studied compound in cannabis after Δ-9-THC, may modulate reward, decisional and sensorimotor processes, being a viable treatment for behavioral addictions.

However, effects of cannabinoids on mental health are far from straightforward. A wide range of effects have been reported over the last decades, from accelerating disease processes to potentially halting them. Graczyk et al. offer an overview of these effects, indicating treatment potential for anxiety, mood, sleep disorders, and addiction, as well as detrimental consequences in terms of psychosis risk and cognitive impairments. Authors link such apparent contrasting evidence to differential effects of cannabis depending on ECS activity, phytocannabinoid composition (Δ-9-THC vs. CBD), terpenoid composition, and dose. Research data from McPherson et al. also indicate an important role of sex in driving the effects of cannabinoids on mental health. More specifically, chronic cannabis use is found to be associated with smaller cerebellum volume and poorer sleep quality, the latter being more pronounced in early-onset cannabis users. Interestingly, females were more sensitive than males to such effects of chronic cannabis use.

Recent years have witnessed a growing interest in the possibility of targeting the ECS to treat major psychiatric disorders. Articles published in this Research Topic are no exception. Cheung et al. address the role of the ECS in neurodevelopment, reviewing the evidence that early cannabinoid treatment may be beneficial under severe conditions such as autism spectrum disorder. To date, CBD has shown the most promising results, also showing a satisfactory safety profile. Also, Cortez et al. review the evidence in support of targeting the ECS in psychosis, beyond the use of antipsychotics aimed at correcting the hyperdopaminergic state seen in the disorder. The authors propose that the cannabinoid receptor type 2 (CB2) may be relevant to different pathophysiological processes observed in psychosis, including not only modulation of dopaminergic neurotransmission, but also microglial activation and stress-induced neuroplastic changes. Again, CBD seems to be a valid treatment also for psychosis, as suggested by Hoffman in his review of preclinical evidence corroborating ECS aberrancies in this disorder and different modulatory effects of CBD on ECS function. Further, Thippaiah et al. discuss the evidence of ECS dysfunction in the context of depression and suicidal behavior, possibly by modulating the hypothalamic-pituitary-adrenal (HPA) axis, neurotrophic factor such as brain derived neurotrophic factor (BDNF), and other neurotransmitters including serotonin, norepinephrine, and dopamine. In his opinion article, Pinna suggests that better characterizing the role of the ECS in mood disorders and comorbid suicidal behavior may result in the identification of more precise neurobiological targets and the related development of novel pharmacological treatments for these conditions.

Finally, Kayser et al. explore in their methods article the translational potential and limitations of human laboratory studies of the effects of cannabinoids. Such studies, especially when implemented with imaging and other neurobiological measures, have helped in modeling addiction, studying the role of cannabinoids in psychiatric disorders, and investigating treatment options. Limited generalizability and participant selection are the main Achilles' heel in these studies. The authors clearly tip the balance in favor of laboratory models as they represent a key translational bridge to inform which preclinical evidence has a chance to result in a successful large-scale clinical study and, possibly, bring a new molecule to the market.

The last decades have seen progressively diminishing numbers of novel drugs between the preclinical and clinical stages of development (7). Translational research must continue to evolve in response to the need to reverse course. Determining and improving the predictive validity of both animal and human laboratory models is one of the challenges of the near future (8). Our hope of developing new medications for psychiatric disorders depends on it.

## Author Contributions

All authors have contributed to the editorial and critically reflected on the successive versions.

## Conflict of Interest

MC has been a consultant/advisor to GW Pharma Limited and F. Hoffmann-La Roche Limited, outside of this work. The remaining authors declare that the research was conducted in the absence of any commercial or financial relationships that could be construed as a potential conflict of interest.

## Publisher's Note

All claims expressed in this article are solely those of the authors and do not necessarily represent those of their affiliated organizations, or those of the publisher, the editors and the reviewers. Any product that may be evaluated in this article, or claim that may be made by its manufacturer, is not guaranteed or endorsed by the publisher.
